# Musculoskeletal Pain Disorders among Secondary School Saudi Female Teachers

**DOI:** 10.1155/2013/878570

**Published:** 2013-07-22

**Authors:** Magdy A. Darwish, Shatha Z. Al-Zuhair

**Affiliations:** ^1^Department of Family & Community Medicine, College of Medicine, University of Dammam, Eastern Province, P.O. Box 1982, Dammam 31441, Saudi Arabia; ^2^Family Medicine Specialists, Dammam Medical Complex, Ministry of Health, Eastern Province, Saudi Arabia

## Abstract

*Objective*. This study was conducted to estimate prevalence and pattern of musculoskeletal pain disorders among secondary school Saudi female teachers in Al-Khobar area and the psychodemographic and psychosocial factors that may affect them. *Material and Method*. A cross-sectional study was conducted using sample of secondary schools teachers (governmental and private school) in Al-Khobar area, Saudi Arabia (KSA). Data were collected using a structured self-administered questionnaire. *Result*. Prevalence of musculoskeletal pain disorders was 79.17%. Main sites of pain were lower back (63.8%) followed by shoulder (45.4%), neck (42.1%), leg (40.0%), wrist (16.2%), and elbow joint (10.0%). Factors that showed significant relationship were type of school (*p* value 0.038), age (*p* value 0.002), weight (*p* value 0.007), number of children (*p* value 0.006), shoe type (*p* value 0.000), teaching years (*p* value 0.003), and working daily hours (*p* value 0.027). *Conclusion*. Secondary school female teachers showed high prevalence of musculoskeletal pain disorders in most anatomic sites, namely, the back, shoulder, neck, legs, wrist, and elbow joint. Risk factors associated with significant pain were type of school, age, weight, number of children, and number of teaching years.

## 1. Introduction and Literature Review

Pain is the most common symptom of which people complain. The most common cause of pain internationally is musculoskeletal pain disorders [1, 2]. Musculoskeletal disorders (MSDs) are put into different categories according to pain location. One category is upper limb disorders which include any injury or disorder located from fingers to shoulder or the neck. Another category of musculoskeletal pain disorder is lower limb disorders which include injury and disorders from hips to toes. Possibly the most common MSD is back pain [3]. MSDs can affect the body's muscles, joints, tendons, ligaments, and nerves. Most work-related MSDs develop over time and are caused either by the work itself or by the employees' working environment. They can also result from fractures sustained in an accident. Health problems range from discomfort, minor aches, and pains, to more serious medical conditions requiring time off work and even medical treatment. In more chronic cases, treatment and recovery are often unsatisfactory, and the result could be permanent disability and loss of employment [4]. Disorders of the musculoskeletal system represent main cause for absence from occupational work, and musculoskeletal disorders lead to considerable costs for the public health system [5]. Many international studies among school teachers have reported high prevalence of musculoskeletal pain disorders [6–12]. School teachers, in general, have been demonstrated relative to other occupational groups, to report a high prevalence of musculoskeletal disorders [13]. Among different populations studied, it was clear that teachers are at higher risk of developing musculoskeletal pain although prevalence among them was not uniform and ranged between 23.7% and 95.1% [9, 13–18]. Musculoskeletal pain is the main cause of absenteeism and early retirement in this category [19]. Teaching leads to stress which will affect school teaching performance [20]. The work tasks of school teachers often involve significant use of a head down posture, such as frequent reading and marking of assignments [21, 22]. The work of teachers does not only involve teaching students but also preparing lessons, assessing students' work, and participating in different school committees. These may cause teachers to suffer adverse mental and physical health issues due to the variety of job functions [6]. There are different patterns of musculoskeletal diseases among men and women, probably reflecting their segregation in different sectors and jobs [3]. Education is a professional field, and proportion of female teachers is rising in most countries [23]. Differences between working conditions of men and women have been mentioned in different studies, and according to these studies, women were less qualified with low salary and had lower control over work and higher level of demands in comparison to men [24]. The prevalence of musculoskeletal pain disorder is positively associated with female gender [6, 14, 21]. 

The aim of study is to assess musculoskeletal pain disorders among secondary school female teachers in Al-Khobar, eastern Saudi Arabia along with psychodemographic and psychosocial factors that may affect them.

## 2. Methodology

A cross-sectional study was conducted to assess musculoskeletal pain disorders among secondary school female teachers in Al-Khobar area, KSA. Sample was taken by multistages, starting with two categories: governmental and private schools. The total number of female secondary schools in Al-Khobar, eastern Saudi Arabia, is 27 schools (governmental: 15 schools and private: 12 schools). Systematic sampling technique from both governmental and private schools takes every second school from the list provided by Ministry of Education followed by questionnaires being distributed to random sample of 20 teachers from each of the selected schools. The outcome was 8 governmental schools with sample size of 160 teachers and 6 private schools with a sample size of 120 teachers, total sample being 280 female teachers. There were a response of 120 questionnaires from private schools with a response rate of 100% and another 120 questionnaires from governmental schools with a response rate of 75% giving total response rate of 87.5% with a total of 240 respondents. The data were collected using structured self-administered questionnaire which consisted of three parts. The first part of the questionnaire was about the respondent's sociodemographic and psychosocial factors and included age, weight, marital status, educational level, number of children, sleeping hours, salary, and characteristic of teaching variables including teaching experience years, working daily hours, number of classes, average number of students per class, and weekly schedule. The second part of the questionnaire investigated the pattern of musculoskeletal pain problems. Site of pain was determined using Nordic musculoskeletal questionnaire (NMQ). Standardized Nordic questionnaire is used for the analysis of musculoskeletal symptoms in an occupational health context. The questions are forced choice variants and may be either self-administered or used in interviews. NMQ is repeatable and sensitive screening and surveillance tool [25–27]. Specific characteristics of work strain are reflected in the third part of the questionnaire to assess the severity of musculoskeletal pain. The Örebro Musculoskeletal Pain Questionnaire (ÖMPSQ) which is a screening questionnaire used to predict long-term disability and failure to return to work in cases of musculoskeletal pain. ÖMPSQ has been used and validated in several studies in acute pain problems. It has been shown to have discriminative power even for patients with nonacute or recurrent pain problems. Indeed, the ÖMPSQ showed better predictive power than any of the relevant similar questionnaires in nonacute and recurrent pain. Cut-off score of 105 has been found to predict those who will have no disabling pain (with 95% accuracy) and those who will have disabling pain (with 67% accuracy) [28–30].

Both questionnaires were translated to Arabic, validated, and modified in light of pilot study. Questionnaires were translated by researchers, one of whom has Saudi Arabian slang, reviewed by 2 faculty, one of whom mastering English language, back translated by different nonmedical expert translator and comparisons were made to ensure same wording and meaning of the questions, and reviewed by researchers before and after pilot study with minor linguistic modifications of some confusing words.

The participants were met, the questionnaires were distributed and explained to them after obtaining a verbal consent, and then questionnaires were collected after being completed. A pilot study was conducted on 20 teachers—different from the target group—to check understanding and clarity of the questionnaire. Based on the results, some linguistic modifications of questions were made to avoid confusion about questions and make easier understanding and interpretation by participants. The data were entered and analyzed in a personal computer using statistical package for social sciences (SPSS) software version 16. Data were presented using descriptive statistics in form of frequencies and percentages for qualitative variables and mean and standard deviation (SD) for quantitative variables. Chi-square test was used as appropriate to determine association. The level of statistical significance was set to be less than 0.05. The study was approved by ethical committee of Postgraduate Saudi Board Program, Eastern Province. Verbal consent was obtained from the participants after explaining objectives of the study to them. All questionnaires were anonymous, and collected data were kept confidential and not used except for the study purpose.

## 3. Results

A total of 240 secondary school female teachers (50% governmental and 50% private schools) participated in the study with a total response rate 87.5%. The sample showed mean age of 35.5 ± 7.8, with age ranging from 22 and 54 years. Majority of the sample of secondary school female teachers were married (64.1%). All of them were university graduates and above. The study showed most of secondary school female teachers to have salary more than 2000 Saudi Riyals (SR) (88.8%) (Table 1). Weight of study sample ranged between 70 and 89 kg in 54% of teachers, while it ranges between 50 and 69 kg in 41.7%. Majority has 1 to 4 children (55.8%). About half of target study teachers (50.8%) have sleeping hours ranging between 6 and 7 hours. Teachers who wear high heel shoes were around (54.6%) (Table 2). Number of teaching years was mainly 1–9 years (48.3%), while in 35.8% it was between 10 and 19 years. Almost 76.7% of sample teachers has 1–4 classes/day, where those who had 5–7 classes per day represent 23.3%. Most of them have 10 to 19 classes/week (59.2%). Those who have from 20 to 30 classes per week were 34.6%. Working daily hours of majority of study group range between 7 and 9 hours (88.8%). Around 62.9% of research sample have 20–34 students per class, while 30.4% have a range between 35 and 50 students (Table 3). The overall prevalence of musculoskeletal pain disorders among 240 study sample was 79.2%. The majority have musculoskeletal pain for more than six months (56.3%). The majority of affected teachers (57.9%) were suffering from multiple sites of musculoskeletal pain. Pattern of musculoskeletal pain among target population showed that highest prevalence was lower back pain (63.8%) followed by shoulder (45.4%), neck pain (42.1%), then leg pain (40.0%), and wrist pain (16.2%), and least one was elbow joint pain which was only (10.0%). The average days of absenteeism were 1.76 ± 2.2 days with significant relationship of number of absence days to Örebro musculoskeletal pain score (*p* value 0.015). Among 240 research sample teachers, 112 (46.7%) have Örebro musculoskeletal pain score Less than 105, of whom 50 (20.8%) have no pain and 62 (25.9%) have nondisabling pain, while those who have Örebro musculoskeletal pain score of 105 and more (disabling pain) were 128 (53.3%). This means that among 190 teachers who reported musculoskeletal pain disorders, about one-third (32.6%) had the Örebro musculoskeletal pain score less than 105 which is considered non disabling pain, while about two-thirds (67.4%) have score 105 and more which is considered disabling pain (Table 4). The study showed significant relationship between Örebro musculoskeletal pain score and type of secondary schools, and disabling musculoskeletal pain was higher among governmental school female teachers (*p* value 0.038). Also increasing age was associated with higher scores (*p* value 0.002), while marital status and salary did not show significant relationship with score. There was significant relationship between Örebro musculoskeletal pain score and increasing weight (*p* value 0.007), number of children (*p* value 0.006), type of shoes (*p* value 0.000), and number of teaching years (*p* value 0.003). Sleeping hours per day (*p* value 0.288), number of classes per day (*p* value 0.206), classes per week (*p* value 0.075), and students per class (*p* value 0.180) did not show significant relationship with Örebro musculoskeletal pain score. Working daily hours of target sample (*p* value 0.027) and duration of musculoskeletal pain (*p* value 0.000) had strong relation with Örebro musculoskeletal pain score where higher scores were associated with chronic, more than six months pain. Higher Örebro musculoskeletal pain scores were significantly associated with increasing days of absenteeism (*p* value 0.015) (Table 5). Multiple logistic regression analysis showed that the following factors are independently and significantly associated with higher Örebro musculoskeletal pain score: weight (OR: 1.755, CI: 1.121–2.748) (*p* value 0.014), working daily hours (OR: 2.758, CI: 1.009–7.543) (*p* value 0.048), and shoes type (OR: 6.411, CI: 3.521–11.673) (*p* value 0.000) (Table 6). 

## 4. Discussion

 In this study the prevalence of musculoskeletal pain was 79.17% among secondary schools female teachers.

 The most prevalent body site on which teachers reported pain was the back (63.8%). This was relatively high figure compared to similar studies. In Philippine and Brazilian studies, 53.3% of secondary school teachers [31] and 41.1% of primary and secondary school teachers [13] have reported back pain, respectively. Again 40.4% of Malay teachers [15] and 40% of Chinese primary school teachers also reported back pain in the 12 months prior to the study [17]. In study about French school teachers, 34.8% of them had experienced back pain in the previous six months [18].

Next most frequent site of musculoskeletal pain reported after back pain was shoulder pain (54.4%) and neck pain (42.1%). These figures were better than what has been reported in many literature. In a study of secondary school teachers in Hong Kong, the life-long prevalence of neck pain has been reported as 69.3% [21]; similar findings have been demonstrated in another Chinese study where secondary school teachers reported a life-long prevalence of neck pain as 68.2% [31]. In a more recent Chinese study, school teachers reported a high neck pain prevalence rate of 68.9% in the previous month [6]. In other studies, 42.5% of Turkish school teachers reported having experienced neck pain [14]. The highest shoulder pain prevalence 73.4% for the previous month has been reported by Chinese school teachers [6], while in Turkey, 28.7% of school teachers had experienced pain in the shoulder area [14]. As we know most teachers use a “head down” posture which is significantly associated with neck pain (OR: 2.17, CI: 1.38–2.74), and this may impact on teachers who spend considerable time correcting students' work and preparing for lessons [21]. 

The prevalence of lower extremity pain was (40%) in our study which was similar to other studies. MSDs in the lower extremities have been reported by 41.1% in Brazilian school teachers [13]. In China, 54.6% of school teachers reported having experienced leg pain during physical activity in the previous month [6]. In a recent Turkish study, lower extremity pain had been experienced by 8.4% of teachers in the hip area, 32% in the knees, and 21.8% in the ankles [24].

Approximately 16.2% reported pain in the wrist and 10% in the elbow joint in our study. This was relatively better than what has been reported in similar studies. Upper limb pain was reported by 23.7% of Brazilian school teachers [13]. In a Chinese study of secondary school teachers, 35.8% reported life-long upper limb pain [21]. Only 8% of school teachers in Turkey reported elbow pain [14]; however, a total of 43.9% of primary and secondary school teachers in Hong Kong reported MSDs in the arm during the previous month [6]. Wrist pain was a symptom reported by only 13% of the Turkish school teachers [14]. 

Musculoskeletal pain is a very complex process influenced by many factors such as sociodemographic characteristics, background characteristics, and work related backgrounds of study sample. 

 In this study there was significant correlation between prevalence of MSDs and age (*p* value 0.002). Musculoskeletal pain disorders are likely to become more prevalent as the population ages throughout the world [32], and this may explain the high prevalence of musculoskeletal pain among the governmental school teachers since 46.7% of governmental school teachers are over 40 years, while only 15% of private secondary schools teachers are above 40 years. This agrees with studies about Brazilian [13] and Turkish teachers [14] which showed that teachers above 40 years were more likely to report MSDs. In other studies, however, younger teachers have also been found to experience MSDs. This has been evidenced in the results of a Chinese study [21] where the age group with the highest prevalence of neck pain was 31–35 years [21]. In another Chinese study, teachers aged 30–39 years had experienced the most low back pain [17]. 

In this study, there was significant correlation between musculoskeletal pain disorders and increasing number of teaching years (*p* value 0.003). This in agreement with results of Chinese secondary school [31] and Brazilian teachers [13]. 

Other sociodemographic factors such as marital status, education level, and salary per months did not show significant correlation to musculoskeletal pain in this study. Musculoskeletal pain disorder showed significant correlation with weight of teachers as this study showed (*p* value 0.007). Increasing the weight of secondary school female teachers was associated with more pain. The association of musculoskeletal pain with obesity has been reported around the world [33]. In both sexes, self-reported work-restricting pain in the neck, back area, hip, knee, and ankle joints was more common in obese subjects than in a general Swedish population (ORs ranging from 1.7 to 9.9, *p* < 0.001) [33]. 

Significant relationship between number of children of the teachers and the musculoskeletal pain was also reported in this study (*p* value 0.006). The teachers of Salvador with three or more children reported more MSDs than those with one or two children [13], and this may be related to more time dedicated to taking care of children, possibility of more psychological stress, and need of a higher work load to increase family income [34]. 

On the other hand number of sleeping hours in this study did not show statistically significant relation with MSDs, but wearing shoes with high heel showed strongly positive relation with musculoskeletal pain disorders (*p* value 0.000). This may be due to disrupting gait and posture for the entire body causing severe load on muscles and ligaments. The number of classes per day and number of students per class did not show significant relation with MSDs. 

 Finally the days of absenteeism have been positively associated with higher Örebro musculoskeletal pain score (*p* value 0.015). As the study done in Natal, Brazil, the musculoskeletal pain was the main cause of absenteeism in school teachers of Natal [19]. 

## 5. Conclusions and Recommendations

Secondary school female teachers showed high prevalence of musculoskeletal pain disorders (79.17%). More than half (53.3%) of those suffering pain were considered significant/disabling and were associated with more days of absenteeism. Modifiable personal and environmental factors provide the opportunity to apply appropriate interventions to reduce the risk of long-term disability. Measures to decrease high prevalence of MSDs among teachers should be implemented to improve their status and avoid harmful and poor impact on their personal and work productivity. These include measures at different levels to see if these measures will help in reducing MSDs. Measures at teacher's level, for example. Health education and promotion programs aiming to encourage maintaining ideal weights and wearing flat medical shoes. Measures at school level, for example, proportional reduction of workload for aging teachers, optimizing working hours per day, planning exercise sessions and ergonomic classes to teach how to avoid/decrease MSDs.

## Figures and Tables

**Table 1:** Sociodemographic characteristics of study sample teachers.

Variable	Total (*n* = 240) (%)
Type of school	
(1) Governmental	120 (50%)
(2) Private	120 (50%)
Age	
(1) (20–<30)	59 (24.6%)
(2) (30–<40)	107 (44.6%)
(3) (40–<50)	59 (24.6%)
(4) (50–<60)	15 (6.2%)
Marital status	
(1) Single	65 (27.1%)
(2) Married	154 (64.2%)
(3) Divorced	15 (6.2%)
(4) Widow	6 (2.5%)
Education level	
(1) University and above	240 (100%)
Salary—Saudi Riyal (SR)	
(1) Equal or less than 2000 SR	27 (11.2%)
(2) More than 2000 SR	213 (88.8%)

**Table 2:** Background characteristics of study sample teachers.

Variable	Total (*n* = 240) (%)
Weight	
(1) (30–<50)	9 (3.8%)
(2) (50–<70)	100 (41.7%)
(3) (70–<90)	108 (45.0%)
(4) (90–<160)	23 (9.6%)
Number of children	
(1) (0)	69 (28.8%)
(2) (1–4)	134 (55.8%)
(3) (5–7)	37 (15.4%)
Sleeping hours	
(1) (4–5)	44 (18.3%)
(2) (6–7)	122 (50.8%)
(3) (8–15)	74 (30.8%)
Shoes	
(1) Flat	109 (45.4%)
(2) With heel	131 (54.6%)

**Table 3:** Work related characteristics of study sample teachers.

Variable	Total (*n* = 240) (%)
Teaching years	
(1) (1–<10)	116 (48.3%)
(2) (10–<20)	86 (35.8%)
(3) (20–<30)	38 (15.8%)
Classes per day	
(1) (1–4)	184 (76.7%)
(2) (5–7)	56 (23.3%)
Classes per week	
(1) (1–9)	15 (6.2%)
(2) (10–19)	142 (59.2%)
(3) (20–30)	83 (34.6%)
Daily hours	
(1) (3–6)	27 (11.2%)
(2) (7–9)	213 (88.8%)
Students per class	
(1) (5–19)	16 (6.7%)
(2) (20–34)	151 (62.9%)
(3) (35–50)	73 (30.4%)

**Table 4:** Pattern and effect of musculoskeletal pain of study sample teachers.

Variable	Total (*n* = 240) (%)
Musculoskeletal pain	
(1) Yes	190 (79.2%)
(2) No	50 (20.8%)
Duration of pain	
(1) No pain	50 (20.8%)
(2) Less than 3 months	31 (12.9%)
(3) From 3 to 6 months	24 (10.0%)
(4) More than 6 months	135 (56.3%)
Areas of the pain	
(1) No pain	50 (20.8%)
(2) Single	51 (21.2%)
(3) Multiple	139 (57.9%)
Site	
(1) No pain	50 (20.8%)
(2) Neck pain	101 (42.1%)
(3) Shoulder pain	109 (45.4%)
(4) Elbow pain	24 (10.0%)
(5) Wrist pain	39 (16.2%)
(6) Low back pain	153 (63 .8%)
(7) Leg pain	96 (40.0%)
(8) Other sites	0 (0.0%)
Days of absenteeism	
(1) (0–5)	227 (94.6%)
(2) (6–10)	12 (5.0%)
(3) (11–15)	1 (0.4%)
Örebro musculoskeletal pain score	
(1) Less than 105	112 (46.7%)
(i) No pain	50 (20.8%)
(ii) Nondisabling pain	62 (25.9%)
(2) 105 and more (disabling pain)	128 (53.3%)

**Table 5:** Relation of different sample variables and Orebro musculoskeletal pain score of study sample teachers.

Variable	No or nondisabling pain (score: less than 105)	Disabling pain (score: 105 and more)	*p* value
Type of school			
(1) Governmental	48 (40.0%)	72 (60 .0%)	0.038
(2) Private	64 (53.3%)	56 (46.7%)	
Age			
(1) (20–29)	40 (67.8%)	19 (32.2%)	0.002
(2) (30–39)	44 (41.1%)	63 (58.9%)	
(3) (40–49)	21 (35.6%)	38 (64.4%)	
(4) (50–60)	7 (46.7%)	8 (53.3%)	
Weight			
(1) (30–49)	5 (55.6%)	4 (44.4%)	0.007
(2) (50–69)	59 (59.0%)	41 (41.0%)	
(3) (70–89)	41 (38.0%)	67 (62.0%)	
(4) (90–160)	7 (30.4%)	16 (69.6%)	
Number of children			
(1) (0)	39 (56.5%)	30 (43.5%)	0.006
(2) (1–4)	64 (47.8%)	70 (52.2%)	
(3) (5–7)	9 (24.3%)	28 (75.7%)	
Shoes			
(1) flat	76 (69.7%)	33 (30.3%)	0.000
(2) With heel	36 (27.5%)	95 (72.5%)	
Teaching years			
(1) (1–9)	67 (57.8%)	49 (42.2%)	0.003
(2) (10–19)	29 (33.7%)	57 (66.3%)	
(3) (20–30)	16 (42.1%)	22 (57.9%)	
Daily hours			
(1) (3–6)	18 (66.7%)	9 (33.3%)	0.027
(2) (7–9)	94 (44.1%)	119 (55.9%)	
Duration of pain			
(1) Less than 3 months	21 (67.7%)	10 (32.3%)	0.000
(2) From 3 to 6 months	8 (33.3%)	16 (66.7%)	
(3) More than 6 months	33 (24.4%)	102 (75.6%)	
Days of absenteeism			
(1) (0–5)	111 (48.9%)	116 (51.1%)	0.015
(2) (6–10)	1 (8.3%)	11 (91.7%)	
(3) (11–15)	0 (0.0%)	1 (100%)	

**Table 6:** Regression analysis of relation of significant factors predicting musculoskeletal pain score among study sample teachers.

Variable	B	S.E	*p* value	EXP (B)	95 C.I. for EXP (B)	
					Lower	Upper
Weight	0.563	0.229	0.014	1.755	1.121	2.748
Daily hours	1.015	0.513	0.048	2.758	1.009	7.543
Shoes type	1.8	0.306	0.000	6.411	3.521	11.673
Constant	−6.387	1.73				

*X*2 (8) = 65.557, *p* < 0.001.
